# Data resource profile: the allergic disease database of the Korean National Health Insurance Service

**DOI:** 10.4178/epih.e2021010

**Published:** 2021-01-21

**Authors:** Sunyong Yoo, Dong-Wook Kim, Young-Eun Kim, Jong Heon Park, Yeon-Yong Kim, Kyu-dong Cho, Mi-Ji Gwon, Jae-In Shin, Eun-Joo Lee

**Affiliations:** 1 Big Data Department, National Health Insurance Service, Wonju, Korea; 2 Department of ICT Convergence System Engineering, Chonnam National University, Gwangju, Korea; 3 Department of Benefits Strategy, National Health Insurance Service, Wonju, Korea; 4 Chungbuk National University Hospital, Cheongju, Korea

**Keywords:** Allergic rhinitis, Atopic dermatitis, Asthma, Database, National health programs

## Abstract

Researchers have been interested in probing how the environmental factors associated with allergic diseases affect the use of medical services. Considering this demand, we have constructed a database, named the Allergic Disease Database, based on the National Health Insurance Database (NHID). The NHID contains information on demographic and medical service utilization for approximately 99% of the Korean population. This study targeted 3 major allergic diseases, including allergic rhinitis, atopic dermatitis, and asthma. For the target diseases, our database provides daily medical service information, including the number of daily visits from 2013 and 2017, categorized by patients’ characteristics such as address, sex, age, and duration of residence. We provide additional information, including yearly population, a number of patients, and averaged geocoding coordinates by *eup*, *myeon*, and *dong* district code (the smallest-scale administrative units in Korea). This information enables researchers to analyze how daily changes in the environmental factors of allergic diseases (e.g., particulate matter, sulfur dioxide, and ozone) in certain regions would influence patients’ behavioral patterns of medical service utilization. Moreover, researchers can analyze long-term trends in allergic diseases and the health effects caused by environmental factors such as daily climate and pollution data. The advantages of this database are easy access to data, additional levels of geographic detail, time-efficient data-refining and processing, and a de-identification process that minimizes the exposure of identifiable personal information. All datasets included in the Allergic Disease Database can be downloaded by accessing the National Health Insurance Service data sharing webpage (https://nhiss.nhis.or.kr).

## INTRODUCTION

Amid increasing global efforts to understand the effects of environmental factors associated with allergic diseases (e.g., environmental pollution, climate change, and harmful substances) on human disease and health, Korea is also seeking to develop effective policies to manage allergic diseases. The increasingly severe problems posed by air pollution, such as fine particulate matter (PM) and yellow dust, have prompted numerous researchers to study the effects of these pollutants on morbidity and mortality. Studies have reported that air pollution and other environmental factors not only increase the prevalence of cancer and cardio-cerebrovascular diseases, but also have impacts on premature deaths and the use of medical services [1-3].

Traditionally, health was defined as a disease-free state, and the medical community focused on treating diseases from a biological perspective. Since the 1970s, the increasing frequency of chronic and lifestyle-related diseases led researchers to acknowledge the power of lifestyle factors as a determinant of health, spurring efforts to prevent diseases based on an improved understanding of the various factors that determine health [4]. Since the late 1980s, an increasing emphasis has been placed on the importance of healthcare policies for managing physical factors at the society-wide and environmental levels, reflecting the awareness that individual efforts are not sufficient to prevent disease and to maintain health [5]. In recent years, disease outbreaks and health equality problems caused by various factors in the physical environment (e.g., climate change and environmental pollution) have become important social issues, along with the social environment. These developments have sparked interest in related research. For instance, the World Health Organization has reported that one-fourth of deaths are caused by preventable environmental factors of allergic diseases, with air pollution (e.g., PM, sulfur dioxide [SO_2_], and ozone [O_3_]) having an especially large effect.

Generally, it has been easy to acquire data in Korea for research purposes through the National Health Insurance Database (NHID), which contains records from the only medical insurance program in the country (provided as a form of social insurance) [6]; however, demand has emerged for additional datasets to use for research into environmental factors of allergic diseases. Multiple studies have investigated these issues, although researchers have specifically pointed out the need for a reliable database that would not require repeated processing and would be suitably constructed for aligning data sets relevant to environmental factors. Researchers have been especially interested in probing how environmental factors affect the use of medical services, as well as the prevalence of death and diseases, and the most common way to analyze big data has been to explore how daily changes in environmental factors in certain regions influence patterns of medical service utilization [7-10]. Considering this demand, we have constructed a database, which we have named the Allergic Disease Database, containing values broken down on the level of district and day to reliably align the National Health Insurance Service (NHIS) big data with data on other environmental factors of allergic diseases.

## DATABASE DESCRIPTION

### Database design

The Allergic Disease Database was established using the NHID, which is an open resource of personally unidentifiable data specifically processed for research purposes. The database contains information on daily medical service utilization, yearly population and number of patients, address codes, and averaged geocoding coordinates by district (Table 1). The daily medical service utilization data comprise a statistical table of daily outpatient visits, inpatient visits, and emergency medical visits by patients’ residential district and basic characteristics (sex, age, and duration of residence). The data on the yearly population and number of patients consist of the number of residents (as of December each year) and the number of patients by disease, residential district, and patients’ basic characteristics (sex, age, and duration of residence). The address and average geocoding coordinates data include address codes from the Ministry of the Interior and Safety, the effective date of address codes, as well as the averaged geocoding coordinates (X, Y) by district. Daily medical service utilization data were calculated based on the serial number of addresses, while the data on the number of population and patients, as well as the address and resident average coordinates, were compiled based on address codes from the Ministry of the Interior and Safety, and should be analyzed with a primary key to understand the relationship between address codes and address serial numbers.

### Baseline population

The NHID has gathered information from 2002 to the present, and includes socio-demographic information and medical service use data from all Korean residents. The current version of the database is built on 5-year data from 2013 to 2017, and information from 2002 onwards will be added to the database on an ongoing basis. We considered people who had health insurance during this period, accounting for 99% of all Koreans. The collected data sets mainly include NHIS subscriber information and diagnosis and treatment history. Korea has adopted a single-provider health insurance system administered by the NHIS, in which all medical service providers and residents are mandated to participate. The single insurer (NHIS) collects individuals’ basic socio-demographic characteristics such as sex, age, residential address, birth, death, and disability status, as well as insurance type and premium data. All medical service providers in Korea file claims for reimbursements through the NHIS, which allows the insurance provider to collect patients’ personal information and data on the provider, service start date, diagnosis code, cost of service, and type of care.

### Structure and composition of the database

In order to build the Allergic Disease Database, we first extracted individual IDs, the number of claims, service start date, and diagnosis code variables from the claims database of the NHID. The variables of residence area, sex, age group, and residence period were extracted from the eligibility database of the NHID. We then integrated information based on the individual ID and grouped the integrated information based on the serve start date, address serial number, sex, age group, duration of residence. Next, statistical values for medical use (e.g., outpatient/inpatient visits, and emergency medical visits) were calculated. Finally, the daily medical service utilization data consist of 15 tables, divided into asthma, atopic dermatitis, and allergic rhinitis disease groups by year from 2013 to 2017. Each table has about 130 million rows and consists of 8 columns, including the total number of populations by region and socio-demographic characteristics, as well as address codes. The yearly population and number of patients consists of 5 tables for each year from 2013 to 2017. Each table has about 100,000 rows and consists of 10 columns, including information on medical utilization by patients’ characteristics based on the date and the region. Lastly, the address information and averaged geocoding coordinates by district are presented in 1 table, with a total of 3,679 rows. The layout is provided in Supplementary Material 1.

### Suggested outcome variables and their distribution

#### Variables at the personal level

There are many ways to define allergic disease groups and subjects in the NHID. This study selected the asthma, atopic dermatitis, and allergic rhinitis disease groups as major allergic diseases, in accordance with the “General guidelines for classification of disease codes and procedure codes for statistical analyses” published by the NHIS [11]. The number of patients varied considerably depending on how many subdiagnoses were included. In 2017, the medical utilization rate of each disease group relative to the total population when including the main diagnosis only, the main and secondary diagnoses, and the main diagnosis and all subdiagnoses were 2.9%, 5.7%, and 11.4%, respectively, for asthma; 1.8%, 2.9%, and 4.7%, respectively, for atopic dermatitis; and 13.1%, 36.9%, and 51.3%, respectively, for allergic rhinitis (Supplementary Material 2). This database was constructed using both the main diagnosis and all subdiagnoses to cover as many patients as possible related to the disease. This is because the classification of high-dimensional statistical data is usually not amenable to standard pattern recognition techniques because of an underlying small sample size problem [12]. The target medical services were diagnosis codes for asthma (J45, J46), atopic dermatitis (L20), and allergic rhinitis (J30) at all medical institutions (medical, public health organizations, and psychiatrists), except visits to oriental medicine providers and dentists, visits with an excluded diagnosis, and visit with missing address information.

#### Repeated variables

**Figure 1.**
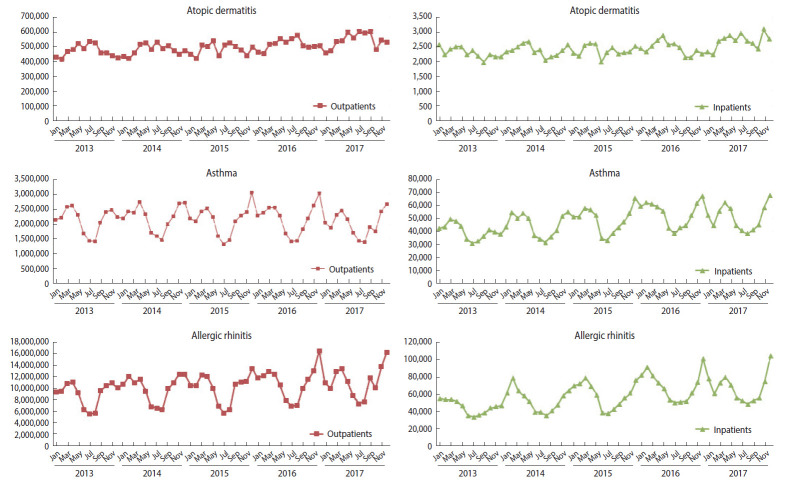
The trends in the sum of monthly outpatient visits and the sum of inpatient visits based on the date of admission between 2013 and 2017.

Instances of medical service utilization included daily outpatient, inpatient, and emergency visits. The number of inpatient visits was calculated using the concept of “episodes,” according to which readmission within 1 day of discharge was considered as the same visit, as this concept is often used to adjust for installment bills in cases of long-term hospitalization. The number of visits during hospitalization was counted in 2 ways: counting the admission date only, and counting every day as a visit over the entire period. Emergency visits were counted as cases billed under the emergency care management fee (Insurance number code: AC101-AC105, V1100-V1400). The trends in the sum of monthly outpatient visits and the sum of inpatient visits based on the date of admission between 2013 and 2017 can be found in Figure 1. The total number of patients in the population by year and in 2017 by sex, age, duration of residence, and day of the week is presented in Table 2.

#### Population-level variables

The yearly population and the number of patients were counted based on individuals with eligibility data as of December in each year, excluding those without detailed address data. The number of patients was counted for inpatient, outpatient, and emergency visits by district (*eup*, *myeon*, and *dong*; the smallest-scale administrative units in Korea) and socio-demographic characteristics (sex and age), removing overlapping patients who had the same main diagnosis code. The daily number of visits for hospital service utilization is listed in Table 3.

### Suggested explanatory variables

#### Multi-level variables

##### Addresses and averaged geocoding coordinates

In the address and averaged geocoding coordinate dataset, new serial numbers for addresses were assigned after integrating the effective date of address codes, cases where the address code changed within a single district, and districts with very small populations that would make personal data identifiable. This dataset can be used as a mapping table to identify the address serial numbers for medical service utilization data. The averaged geocoding coordinate value data can be used to calculate distance values when aligned with data from different institutions.

The address code serial numbers were set by assigning the same number for each integrated district. The requirements for integration were as follows: districts that went through any changes (creation, deletion, division, integration) at least once between 2013 and 2017 were integrated under the same number, using the “Resident registration address code change history” at the Ministry of the Interior and Security webpage (http://www.mois.go.kr). Districts with an extremely small number of residents were integrated with nearby administrative units and were assigned the same serial number to prevent the identification of personal data. Districts that had fewer than 5 individuals in any category at any point between 2013 and 2017 by district, sex, and age were selected as eligible for de-identification and were assigned a serial number after integration with nearby administrative units. A total of 351 district-level units were integrated (Supplementary Material 3).

#### Variables at the personal level

The daily medical service utilization dataset contains the number of daily visits between 2013 and 2017 broken down by patients’ characteristics (address, sex, age, and duration of residence), including figures for each day of the week. The characteristics of patients were determined by referencing the information recorded in the eligibility data for the month of medical service use (e.g., December 2012 eligibility data were referenced when a patient used a service in December 2012), sex was defined as male and female, and age was grouped in 5-year intervals. Duration of residence was defined as the duration of residence in a certain region over the past 10 years based on the address data for the applicable months. For example, if a patient lived for 9 years and 8 months in address code A and for 4 months in address code B, the utilization of medical services in address code A would fall into category 3 (5 years or longer), while the utilization of medical services in address code B would fall in category 1 (1 year or less).

## RESEARCH APPLICATION

### Prior data resource use

Previous studies on allergic diseases using NHIS claim data have mainly used generalized additive models that refined the claims data into data on the daily number of visits for outpatient, inpatient, and emergency visits, which were aligned with air pollution data from the Urban Air Monitoring Network [7,9]. Factors influencing health and air pollution data (PM with aerodynamic diameter up to 10 μm, O_3_, nitrogen dioxide [NO_2_], SO_2_, and carbon monoxide) were most commonly used as analysis variables, while factors such as day of the week, climate variables, and long-term trends were controlled for using locally weighted regression smoothing functions. However, the results varied depending on target selection (e.g., when analyses were limited to children, the elderly, certain regions or diagnosis codes, or diagnosis and pharmaceutical codes), and analyses of inpatient cases showed variation according to the criteria used to calculate the number of inpatient episodes and the selection of lag effect dates.

In previous studies on factors affecting atopic dermatitis, Lee et al. [9] found no statistically significant variables, while Kim et al. [8] demonstrated that O_3_ affected the number of outpatient visits and fine PM significantly affected the number of outpatient visits only in some regions. For asthma, Lee et al. [9] concluded that fine PM, O_3_, and NO_2_ affected the number of patients while Kim et al. [8] reported that O_3_ meaningfully affected outpatient visits and that O_3_ and fine PM meaningfully influenced the number of inpatients in some regions. Many studies have concluded that fine PM levels affected asthma [9], although Kim et al. [8] concluded that O_3_ meaningfully affected the number of outpatients in all metropolitan areas across the country, whereas fine PM only affected the number of outpatients and inpatients in some regions.

### Further application

The proposed Allergic Disease Database can be used to predict future allergic disease occurrence and regional medical use through time series analysis [13,14]. It can be used to support the design of effective public health policies to prevent allergic diseases and health promotion. It will also be possible to conduct specific forms of longitudinal studies with sample cohorts, performing cross-sections at intervals over time based on socio-demographic and allergic disease information contained in the database [15,16]. In addition, the database can be used to conduct further analyses by combining various external data. By adding environmental parameters (e.g., PM, yellow dust, and SO_2_) to the database, it will be possible to analyze the transient effects of those parameters on allergic disease risk by conducting case-crossover studies [17-19].

## STRENGTHS AND WEAKNESSES

The Allergic Disease Database has the following strengths: First, its data can be easily accessed by simply logging onto the webpage, without having to apply for access to the data. Second, it is possible to break down the results by small-scale address units. The Allergic Disease Database provides addresses down to the district level (*eup*, *myeon*, and *dong*), while other data sets shared or provided upon researchers’ request normally provide addresses only at the city, province, and county level. Third, the database saves time for data refining and processing. The accuracy of the address codes has been improved by refining the data with effective address codes in the applicable year, and the adjusted and claimed data were transformed into daily data by accounting for address code changes. Outpatient visit data were refined by episodes to reduce the work needed for data refinement and to utilize the data efficiently. Fourth, the database provides results for different types of medical service utilization, including outpatient, inpatient (by admission start date and duration of admission), and emergency care. Fifth, there is a reduced risk of personal identification through de-identification. Although the database was not subject to de-identification guidelines due to its form as a statistical data, the risk of identification was minimized by grouping together districts with fewer than 5 residents in any category. Lastly, it is possible to explain the characteristics of allergic disease factors over time by analyzing the pattern of seasonal fluctuations in the distribution of disease prevalence. We plan to support more diverse analyses by expanding the target diseases to include cardiovascular diseases and infectious diseases affected by the environment.

The acknowledged limitations of this database are as follows: First, it is difficult for researchers to perform a customized analysis because the information provided in the database is in the form of statistical values calculated based on a predefined operational definition. In addition, since the allergic diseases currently under consideration are chronic, there is a limitation in evaluating the incidence rate only by analyzing utilization of medical services. In order to solve this problem, operational definitions should be subdivided according to the characteristics of the disease. Therefore, it is necessary to define and update operational definitions in consideration of drugs and treatments for specific diseases in consultation with academic researchers and experts in the future. Second, the risk of type I error (false positive) is considerable, because the main diagnosis and all subdiagnoses were considered when extracting the disease group. Type I error refers to a situation in which the test result erroneously indicates the presence of the disease, whereas type II error (false negative) refers to the opposite situation in which the test result does not indicate the presence of the disease. If we only use the main diagnosis, excluding all subdiagnoses, type 1 error decreases but type 2 error increases. In this study, we used all subdiagnoses as it was considered important to reduce type 2 error. However, depending on the researcher’s study design, reducing type 1 error may be more important. Therefore, we plan to conduct further analyses on various subdiagnoses. Third, autocorrelation problems may occur in repeated analyses by region or episode. If researchers do not consider a proper normalization process or multi-level analysis, non-significant results can be distorted into significant results. Fourth, the actual number of individuals affected by these illnesses may be different from the values provided in this database, as the values in this database were calculated based on diagnoses. Fifth, it may not be possible to analyze integrated districts individually.

## DATA ACCESSIBILITY

All datasets included in the Allergic Disease Database can be downloaded by accessing the NHIS data sharing webpage (https://nhiss.nhis.or.kr). Sign up, sign in, and click “Data application” → “Allergic disease DB.” The Allergic Disease Database manual and sample data can be downloaded by clicking “Download the manual and sample data,” and the entire dataset can be downloaded by clicking “Application for data use” after submitting the name of the research director and purpose of research and clicking “Apply.”

All data files are provided in CSV format. There are a total of 15 files, including medical use data by year (5 files) and by disease (3 files), yearly population and number of visits (5 files), and data on address codes and averaged geocoding coordinates (1 file). A detailed user manual can be downloaded at the same time, and any questions can be directed to the NHIS Big Data Office at +82-33-736-2462.

### Ethics statement

The Ethics Committee of the NHIS waived the need to obtain consent for the collection, analysis, and publication of the retrospectively obtained and anonymized data for this non-interventional study.

## Figures and Tables

**Table 1.** Components of the Allergic Disease Dataset

Dataset	Variables
Daily medical service utilization	Grouping of individuals: address serial no.^1^, age, sex, duration of residence
	Values: daily outpatient visits, inpatients visits, emergency medical (EM) visits
Yearly population and no. of patients	Grouping of individuals: address information (address serial no.^1^, address code^2^, road name address), age, sex
	Values: population, no. of inpatients and outpatients, inpatients, outpatients, and EM patients
Address information and averaged geocoding coordinates by district	Grouping of individuals: address information (address serial no.^1^, address code^2^, road name address, effective date of address code)
	Values: averaged geocoding coordinates (X,Y) by district

1 Primary key ①: address serial number.

2 Primary key ②: address code.

**Table 2.** Total number of patients in the yearly population and in the patient dataset

Variables	Year	Inpatients and outpatients	Outpatients	Inpatients	Emergency
Atopic dermatitis (L20)	2013	2,249,182	2,232,866	25,010	6,707
	2014	2,287,569	2,270,475	25,378	7,470
	2015	2,301,699	2,284,560	25,334	7,028
	2016	2,386,864	2,369,496	25,516	6,209
	2017	2,491,947	2,471,877	28,036	6,794
Asthma (J45, J46)	2013	6,089,879	5,951,971	352,489	128,336
	2014	6,482,546	6,323,475	402,240	139,991
	2015	6,453,093	6,270,541	444,186	143,138
	2016	6,637,868	6,436,098	485,559	156,131
	2017	6,166,104	5,965,176	451,392	145,517
Allergic rhinitis (J30)	2013	24,243,255	24,137,972	452,490	134,651
	2014	26,409,392	26,293,309	532,892	181,242
	2015	26,497,667	26,372,444	591,168	190,989
	2016	27,946,912	27,810,667	686,996	264,215
	2017	28,366,850	28,228,215	654,118	262,313

**Table 3.** Total number of visits in the daily medical service utilization dataset (2017)

Variables		Atopic dermatitis (visits)				Asthma (visits)				Allergic rhinitis (visits)			
		Outpatients	Inpatients^1^	Inpatients^2^	EM	Outpatients	Inpatients^1^	Inpatients^2^	EM	Outpatients	Inpatients^1^	Inpatients^2^	EM
Total		6,521,354	32,210	379,293	7,136	24,043,229	607,214	7,474,389	176,809	134,248,550	804,786	6,796,057	301,064
Sex	Male	3,216,508	17,589	220,498	4,019	11,363,496	306,215	3,491,127	97,016	61,483,815	399,894	3,309,206	153,920
	Female	3,304,846	14,621	158,795	3,117	12,679,733	300,999	3,983,262	79,793	72,764,735	404,892	3,486,851	147,144
Age (yr)	0-4	1,281,774	11,554	66,514	1,812	6,597,447	200,089	1,189,263	30,296	27,466,088	244,879	1,373,882	70,401
	5-9	803,083	2,843	17,033	520	3,140,307	41,679	239,381	7,470	17,273,771	71,110	388,512	24,568
	10-14	412,625	1,234	9,231	255	871,463	12,951	78,984	3,041	7,431,696	29,504	170,477	11,470
	15-19	409,269	808	7,226	203	494,842	6,153	44,186	2,448	5,799,677	24,353	140,883	13,450
	20-24	347,972	613	5,350	184	352,209	4,332	33,094	2,417	3,934,096	20,765	113,484	11,948
	25-29	284,509	537	5,695	164	409,987	4,036	32,826	2,407	4,424,748	17,356	99,084	11,667
	30-34	238,395	511	5,872	182	537,817	5,190	43,097	2,472	5,577,867	19,975	125,130	13,087
	35-39	275,641	685	8,270	299	787,424	7,479	69,083	3,207	7,714,854	25,919	183,311	15,680
	40-44	259,383	786	9,817	227	783,871	8,253	87,288	3,817	7,016,780	25,287	206,909	14,281
	45-49	286,240	1,071	14,604	283	865,158	11,263	136,588	5,137	7,003,185	31,212	286,781	15,514
	50-54	275,043	1,312	16,678	306	893,364	15,849	207,033	6,120	6,527,694	38,530	393,943	15,617
	55-59	323,737	1,568	22,098	362	1,236,881	25,510	344,104	8,621	7,784,808	52,210	564,609	19,131
	60-64	294,011	1,626	25,428	363	1,307,772	29,974	419,954	9,740	6,920,502	46,504	530,276	14,705
	65-69	276,644	1,625	32,008	368	1,321,343	32,336	491,803	10,895	5,859,788	37,817	474,523	11,294
	70-74	270,817	1,855	42,741	470	1,353,925	39,007	637,782	14,348	5,041,092	34,016	467,708	10,270
	75-79	245,253	1414	31,231	420	1,514,338	55,510	982,521	21,333	4,640,255	36,963	521,759	11,357
	≥80	236,958	2168	59,497	718	1,575,081	107,603	2,437,402	43,040	3,831,649	48,386	754,786	16,624
Residential duration (yr)	< 1	1,504,590	10,539	89,411	2,074	5,310,728	164,062	1,470,313	36,957	28,855,837	215,349	1,505,476	72,732
	1-4	1,773,715	8,716	85,155	1,696	7,360,981	164,494	1,567,415	37,029	40,556,667	238,504	1,707,633	81,356
	≥5	3,243,049	12,955	204,727	3,366	11,371,520	278,658	4,436,661	102,823	64,836,046	350,933	3,582,948	146,976
Day of the week	Monday	1,355,752	6,807	54,884	1,125	5,170,006	124,715	1,097,571	27,047	28,520,934	169,394	1,008,899	39,617
	Tuesday	1,079,237	5,337	54,501	966	3,761,474	93,995	1,066,885	23,651	20,934,311	125,821	969,502	32,560
	Wednesday	984,431	5,009	54,916	959	3,538,714	93,839	1,076,812	23,325	19,378,533	123,572	984,614	32,758
	Thursday	1,023,497	4,910	55,376	861	3,839,567	88,476	1,080,856	23,005	21,136,031	120,156	994,660	31,156
	Friday	1,091,497	4,601	55,396	913	3,952,706	88,584	1,086,162	23,217	21,884,221	118,690	1,001,299	32,128
	Saturday	897,589	2,996	53,238	1,054	3,190,540	65,327	1,055,038	25,399	18,859,435	83,244	955,366	48,104
	Sunday	89,351	2,550	50,982	1,258	590,222	52,278	1,11,65	31,165	3,535,085	63,909	881,717	84,741

EM, emergency medicine.

1 Inpatients refers to the use of the admission start date as the criterion for defining an inpatient visit.

2 Inpatients refers to the use of admission duration.
