# Supplementary material for: Data resource profile: the allergic disease database of the Korean National Health Insurance Service
Source: Epidemiol Health. 2021 Jan 21;43:e2021010. doi: 10.4178/epih.e2021010 (PMC8060521; doi:10.4178/epih.e2021010)
Supplement: Supplementary Material 1. [file epih-43-e2021010-suppl1.docx]

**Supplementary Material 1. The Allergic Disease Database layout**

1. Daily medical service utilization

| No | Variable | Variable name | Format | Length | Coding value/Primary key |
| --- | --- | --- | --- | --- | --- |
| (1) | DT | Date | varchar | 20 |  |
| (2) | DAY | Day of the weeks | int | 8 | 1: Mon/2: Tue/...7: Sun |
| (3) | **ADDR_NUM** | Address serial number | int | 8 | ***PRIMARY KEY①** |
| (4) | AGE | Age group | int | 8 | 0: 0~4/5 : 5~9 ... 80 : over 80 |
| (5) | SEX_TYPE | sex | int | 8 | 1 : male/2 : female |
| (6) | SYD | Duration of residence | int | 8 | 1 : below 1 year/2 : 1~4 year/  3 :over 5 year |
| (7) | OUT_CNT | No. of Outpatient visits | int | 8 |  |
| (8) | IN_CNT | No. of inpatient visits  (criteria: service start date) | int | 8 |  |
| (9) | IN_CNT2 | No. of inpatient visits  (criteria: service duration) | int | 8 |  |
| (10) | EM_CNT | No. of emergency visits | int | 8 |  |

2. Yearly population and number of patients

| No | Variable | Variable name | Format | Length | Coding value/Primary key |
| --- | --- | --- | --- | --- | --- |
| (1) | **ADDR** | Address code | varchar | 16 | ***PRIMARY KEY②** |
| (2) | **NUM** | Address serial number | int | 8 | ***PRIMARY KEY①** |
| (3) | SD_NM | Address name_Sido | varchar | 100 |  |
| (4) | SGG_NM | Address name_Gungu | varchar | 100 |  |
| (5) | EMD_NM | Address name_eup, myeon, dong | varchar | 100 |  |
| (6) | AGE | Age group | int | 8 | 0: 0~4/5 : 5~9 ... 80 : over 80 |
| (7) | SEX_TYPE | Sex | varchar | 2 | 1 : male/2 : female |
| (8) | CNT | No of residence | int | 8 |  |

3. Address information and averaged geocoding coordinates by district

| No | Variable | Variable name | Format | Length | Coding value/Primary key |
| --- | --- | --- | --- | --- | --- |
| (1) | **ADDR_CD** | Address code | varchar | 16 | ***PRIMARY KEY②** |
| (2) | **NUM** | Address serial number | int | 8 | ***PRIMARY KEY①** |
| (3) | SD_NM | Address name_Sido | varchar | 100 |  |
| (4) | SGG_NM | Address name_Gungu | varchar | 100 |  |
| (5) | EMD_NM | Address name_eup, myeon, dong | varchar | 100 |  |
| (6) | STRT_DT | Address code effective date | varchar | 16 |  |
| (7) | END_ET | Address code expiration date | varchar | 16 |  |
| (8) | AVG_X_COORD | Averaged geocoding X | varchar |  |  |
| (9) | AVG_Y_COORD | Averaged geocoding Y | varchar |  |  |
