# Supplementary material for: Data resource profile: the allergic disease database of the Korean National Health Insurance Service
Source: Epidemiol Health. 2021 Jan 21;43:e2021010. doi: 10.4178/epih.e2021010 (PMC8060521; doi:10.4178/epih.e2021010)
Supplement: Supplementary Material 2. [file epih-43-e2021010-suppl2.docx]

**Supplementary Material 2. Comparison of the number of patients by diagnosis classification (2017)**

|  |  | **Main diagnosis** | | **Secondary diagnosis** | | **Main and subdiagnoses** | |
| --- | --- | --- | --- | --- | --- | --- | --- |
|  |  | **n** | **%**† | **n** | **%**† | **n** | **%**† |
| **Atopic dermatitis (L20)** | 2013 | 1,009,857 | 2.0 | 1,546,361 | 3.0 | 2,216,821 | 4.3 |
|  | 2014 | 977,246 | 1.9 | 1,518,425 | 2.9 | 2,252,712 | 4.3 |
|  | 2015 | 951,793 | 1.8 | 1,486,779 | 2.8 | 2,258,965 | 4.3 |
|  | 2016 | 959,085 | 1.8 | 1,510,966 | 2.9 | 2,361,300 | 4.5 |
|  | 2017 | 963,552 | 1.8 | 1,528,262 | 2.9 | 2,452,180 | 4.7 |
| **Asthma (J45, J46)** | 2013 | 1,915,813 | 3.7 | 3,617,636 | 7.0 | 5,947,364 | 11.5 |
|  | 2014 | 1,868,306 | 3.6 | 3,623,969 | 7.0 | 6,328,649 | 12.2 |
|  | 2015 | 1,745,294 | 3.3 | 3,433,376 | 6.6 | 6,274,147 | 12.0 |
|  | 2016 | 1,721,904 | 3.3 | 3,408,671 | 6.5 | 6,501,264 | 12.4 |
|  | 2017 | 1,540,580 | 2.9 | 3,027,766 | 5.7 | 6,016,929 | 11.4 |
| **Allergic rhinitis (J30)** | 2013 | 6,125,748 | 11.8 | 17,311,689 | 33.4 | 23,407,070 | 45.2 |
|  | 2014 | 6,532,541 | 12.5 | 18,618,773 | 35.8 | 25,405,198 | 48.8 |
|  | 2015 | 6,446,980 | 12.3 | 18,370,648 | 35.1 | 25,413,439 | 48.5 |
|  | 2016 | 6,907,900 | 13.1 | 19,402,527 | 36.9 | 26,964,152 | 51.3 |
|  | 2017 | 7,083,366 | 13.4 | 19,489,122 | 37.0 | 27,282,285 | 51.8 |

† Number of patients/total population (2013: 51,783,096, 2014: 52,073,761, 2015: 52,359,724, 2016: 52,585,445, 2017: 52,712,246)
