# Supplementary material for: Data resource profile: the allergic disease database of the Korean National Health Insurance Service
Source: Epidemiol Health. 2021 Jan 21;43:e2021010. doi: 10.4178/epih.e2021010 (PMC8060521; doi:10.4178/epih.e2021010)
Supplement: Supplementary Material 3. [file epih-43-e2021010-suppl3.docx]

**Supplementary Material 3. Address serial number list**

| **no** | **Address code** | **Address**  **Serial**  **number** | **Address name** | | | **Effective date** | **Expiration**  **date** |
| --- | --- | --- | --- | --- | --- | --- | --- |
| 1 | 11140610 | 26 | 서울특별시 | 중구 | 신당제1동 | 19880423 | 20130720 |
| 2 | 11140615 | 26 | 서울특별시 | 중구 | 신당동 | 20130720 | 99991231 |
| 3 | 11140620 | 28 | 서울특별시 | 중구 | 신당제2동 | 19880423 | 20130720 |
| 4 | 11140625 | 28 | 서울특별시 | 중구 | 다산동 | 20130720 | 99991231 |
| 5 | 11140630 | 30 | 서울특별시 | 중구 | 신당제3동 | 19880423 | 20130720 |
| 6 | 11140635 | 30 | 서울특별시 | 중구 | 약수동 | 20130720 | 99991231 |
| 7 | 11140640 | 32 | 서울특별시 | 중구 | 신당제4동 | 19880423 | 20130720 |
| 8 | 11140645 | 32 | 서울특별시 | 중구 | 청구동 | 20130720 | 99991231 |
| 9 | 11140660 | 35 | 서울특별시 | 중구 | 신당제6동 | 19880423 | 20130720 |
| 10 | 11140665 | 35 | 서울특별시 | 중구 | 동화동 | 20130720 | 99991231 |
| 11 | 11710646 | 405 | 서울특별시 | 송파구 | 장지동 | 19960801 | 99991231 |
| 12 | 11710647 | 405 | 서울특별시 | 송파구 | 위례동 | 20150706 | 99991231 |
| 13 | 26170610 | 462 | 부산광역시 | 동구 | 좌천제1동 | 19950101 | 20150701 |
| 14 | 26170640 | 462 | 부산광역시 | 동구 | 좌천제4동 | 19950101 | 20150701 |
| 15 | 26170645 | 462 | 부산광역시 | 동구 | 좌천동 | 20150701 | 99991231 |
| 16 | 26170650 | 463 | 부산광역시 | 동구 | 범일제1동 | 19950101 | 99991231 |
| 17 | 26170670 | 463 | 부산광역시 | 동구 | 범일제4동 | 19950101 | 20160101 |
| 18 | 26230510 | 478 | 부산광역시 | 부산진구 | 부전제1동 | 19950101 | 99991231 |
| 19 | 26230530 | 478 | 부산광역시 | 부산진구 | 범전동 | 19950101 | 20150101 |
| 20 | 26230600 | 485 | 부산광역시 | 부산진구 | 전포제1동 | 19950101 | 99991231 |
| 21 | 26230620 | 485 | 부산광역시 | 부산진구 | 전포3동 | 19950101 | 20150101 |
| 22 | 26230670 | 490 | 부산광역시 | 부산진구 | 당감제1동 | 19950101 | 99991231 |
| 23 | 26230690 | 490 | 부산광역시 | 부산진구 | 당감제3동 | 19950101 | 20130201 |
| 24 | 26230710 | 494 | 부산광역시 | 부산진구 | 가야제1동 | 19950101 | 99991231 |
| 25 | 26230730 | 494 | 부산광역시 | 부산진구 | 가야제3동 | 19950101 | 20130201 |
| 26 | 26230780 | 501 | 부산광역시 | 부산진구 | 범천제2동 | 19950101 | 99991231 |
| 27 | 26230790 | 501 | 부산광역시 | 부산진구 | 범천제4동 | 19950101 | 20160101 |
| 28 | 26290510 | 516 | 부산광역시 | 남구 | 대연제1동 | 19950101 | 99991231 |
| 29 | 26290520 | 516 | 부산광역시 | 남구 | 대연제2동 | 19950101 | 20130701 |
| 30 | 26290640 | 530 | 부산광역시 | 남구 | 우암제1동 | 19950101 | 20130701 |
| 31 | 26290645 | 530 | 부산광역시 | 남구 | 우암동 | 20130701 | 99991231 |
| 32 | 26290650 | 530 | 부산광역시 | 남구 | 우암제2동 | 19950101 | 20130701 |
| 33 | 26350510 | 551 | 부산광역시 | 해운대구 | 우제1동 | 19950101 | 99991231 |
| 34 | 26350520 | 551 | 부산광역시 | 해운대구 | 우제2동 | 19950101 | 99991231 |
| 35 | 26350525 | 551 | 부산광역시 | 해운대구 | 우제3동 | 20160101 | 99991231 |
| 36 | 26350610 | 563 | 부산광역시 | 해운대구 | 반송제1동 | 19981001 | 99991231 |
| 37 | 26350630 | 563 | 부산광역시 | 해운대구 | 반송제3동 | 19950101 | 20151001 |
| 38 | 26440570 | 608 | 부산광역시 | 강서구 | 천가동 | 19950101 | 20150130 |
| 39 | 26440580 | 608 | 부산광역시 | 강서구 | 가덕도동 | 20150130 | 99991231 |
| 40 | 26710256 | 645 | 부산광역시 | 기장군 | 정관읍 | 20150923 | 99991231 |
| 41 | 26710320 | 645 | 부산광역시 | 기장군 | 정관면 | 19950301 | 20150923 |
| 42 | 28185820 | 843 | 인천광역시 | 연수구 | 송도1동 | 20120101 | 99991231 |
| 43 | 28185840 | 843 | 인천광역시 | 연수구 | 송도3동 | 20140924 | 99991231 |
| 44 | 28245710 | 896 | 인천광역시 | 계양구 | 계양1동 | 19950301 | 99991231 |
| 45 | 28245730 | 896 | 인천광역시 | 계양구 | 계양3동 | 20150401 | 99991231 |
| 46 | 28260536 | 901 | 인천광역시 | 서구 | 청라1동 | 20120709 | 99991231 |
| 47 | 28260537 | 901 | 인천광역시 | 서구 | 청라2동 | 20120709 | 99991231 |
| 48 | 28260539 | 901 | 인천광역시 | 서구 | 청라3동 | 20160701 | 99991231 |
| 49 | 28260630 | 917 | 인천광역시 | 서구 | 검단1동 | 20020101 | 20180701 |
| 50 | 28260670 | 917 | 인천광역시 | 서구 | 검단5동 | 20130902 | 20180701 |
| 51 | 29170695 | 1,011 | 광주광역시 | 북구 | 건국동 | 19980921 | 99991231 |
| 52 | 29170696 | 1,011 | 광주광역시 | 북구 | 양산동 | 20130318 | 99991231 |
| 53 | 30200547 | 1,094 | 대전광역시 | 유성구 | 노은2동 | 20070205 | 99991231 |
| 54 | 30200548 | 1,094 | 대전광역시 | 유성구 | 노은3동 | 20150720 | 99991231 |
| 55 | 30200530 | 1,099 | 대전광역시 | 유성구 | 온천1동 | 19950101 | 99991231 |
| 56 | 30200610 | 1,099 | 대전광역시 | 유성구 | 원신흥동 | 20130916 | 99991231 |
| 57 | 31110560 | 1,125 | 울산광역시 | 중구 | 북정동 | 19970715 | 20140901 |
| 58 | 31110650 | 1,125 | 울산광역시 | 중구 | 성안동 | 20140901 | 99991231 |
| 59 | 36110510 | 1,185 | 세종특별자치시 | ? | 한솔동 | 20120701 | 99991231 |
| 60 | 36110515 | 1,185 | 세종특별자치시 | ? | 새롬동 | 20171016 | 99991231 |
| 61 | 36110520 | 1,185 | 세종특별자치시 | ? | 도담동 | 20140210 | 99991231 |
| 62 | 36110530 | 1,185 | 세종특별자치시 | ? | 아름동 | 20150126 | 99991231 |
| 63 | 36110540 | 1,185 | 세종특별자치시 | ? | 종촌동 | 20160418 | 99991231 |
| 64 | 36110560 | 1,185 | 세종특별자치시 | ? | 보람동 | 20170206 | 99991231 |
| 65 | 41113660 | 1,204 | 경기도 | 수원시 권선구 | 금호동 | 20031124 | 20160111 |
| 66 | 41113662 | 1,204 | 경기도 | 수원시 권선구 | 금곡동 | 20160111 | 99991231 |
| 67 | 41113664 | 1,204 | 경기도 | 수원시 권선구 | 호매실동 | 20160111 | 99991231 |
| 68 | 41117570 | 1,227 | 경기도 | 수원시 영통구 | 영통1동 | 20031124 | 99991231 |
| 69 | 41117580 | 1,227 | 경기도 | 수원시 영통구 | 영통2동 | 20031124 | 99991231 |
| 70 | 41117585 | 1,227 | 경기도 | 수원시 영통구 | 영통3동 | 20171226 | 99991231 |
| 71 | 41117565 | 1,229 | 경기도 | 수원시 영통구 | 광교동 | 20121226 | 20150120 |
| 72 | 41117600 | 1,229 | 경기도 | 수원시 영통구 | 광교1동 | 20150120 | 99991231 |
| 73 | 41117610 | 1,229 | 경기도 | 수원시 영통구 | 광교2동 | 20150120 | 99991231 |
| 74 | 41131620 | 1,244 | 경기도 | 성남시 수정구 | 복정동 | 19890101 | 99991231 |
| 75 | 41131625 | 1,244 | 경기도 | 성남시 수정구 | 위례동 | 20151102 | 99991231 |
| 76 | 41135545 | 1,263 | 경기도 | 성남시 분당구 | 정자동 | 20150501 | 99991231 |
| 77 | 41135550 | 1,263 | 경기도 | 성남시 분당구 | 정자1동 | 20010101 | 99991231 |
| 78 | 41150600 | 1,295 | 경기도 | 의정부시 | 가능2동 | 19920801 | 20170403 |
| 79 | 41150610 | 1,295 | 경기도 | 의정부시 | 가능3동 | 19880423 | 20170403 |
| 80 | 41150615 | 1,295 | 경기도 | 의정부시 | 흥선동 | 20170403 | 99991231 |
| 81 | 41190510 | 1,328 | 경기도 | 부천시 | 심곡2동 | 20160704 | 99991231 |
| 82 | 41195520 | 1,328 | 경기도 | 부천시 원미구 | 심곡2동 | 19930115 | 20160704 |
| 83 | 41190520 | 1,329 | 경기도 | 부천시 | 심곡1동 | 20160704 | 99991231 |
| 84 | 41195510 | 1,329 | 경기도 | 부천시 원미구 | 심곡1동 | 19930115 | 20160704 |
| 85 | 41190530 | 1,330 | 경기도 | 부천시 | 심곡3동 | 20160704 | 99991231 |
| 86 | 41195530 | 1,330 | 경기도 | 부천시 원미구 | 심곡3동 | 19930115 | 20160704 |
| 87 | 41190540 | 1,331 | 경기도 | 부천시 | 원미2동 | 20160704 | 99991231 |
| 88 | 41195550 | 1,331 | 경기도 | 부천시 원미구 | 원미2동 | 19930115 | 20160704 |
| 89 | 41190550 | 1,332 | 경기도 | 부천시 | 소사동 | 20160704 | 99991231 |
| 90 | 41195560 | 1,332 | 경기도 | 부천시 원미구 | 소사동 | 19930115 | 20160704 |
| 91 | 41190560 | 1,333 | 경기도 | 부천시 | 원미1동 | 20160704 | 99991231 |
| 92 | 41195540 | 1,333 | 경기도 | 부천시 원미구 | 원미1동 | 19930115 | 20160704 |
| 93 | 41190570 | 1,334 | 경기도 | 부천시 | 역곡1동 | 20160704 | 99991231 |
| 94 | 41195570 | 1,334 | 경기도 | 부천시 원미구 | 역곡1동 | 19930115 | 20160704 |
| 95 | 41190580 | 1,335 | 경기도 | 부천시 | 역곡2동 | 20160704 | 99991231 |
| 96 | 41195580 | 1,335 | 경기도 | 부천시 원미구 | 역곡2동 | 19930115 | 20160704 |
| 97 | 41190590 | 1,336 | 경기도 | 부천시 | 춘의동 | 20160704 | 99991231 |
| 98 | 41195590 | 1,336 | 경기도 | 부천시 원미구 | 춘의동 | 19930115 | 20160704 |
| 99 | 41190600 | 1,337 | 경기도 | 부천시 | 도당동 | 20160704 | 99991231 |
| 100 | 41195600 | 1,337 | 경기도 | 부천시 원미구 | 도당동 | 19930115 | 20160704 |
| 101 | 41190610 | 1,338 | 경기도 | 부천시 | 중동 | 20160704 | 99991231 |
| 102 | 41195620 | 1,338 | 경기도 | 부천시 원미구 | 중동 | 19930115 | 20160704 |
| 103 | 41190620 | 1,339 | 경기도 | 부천시 | 상동 | 20160704 | 99991231 |
| 104 | 41195670 | 1,339 | 경기도 | 부천시 원미구 | 상동 | 19930115 | 20160704 |
| 105 | 41190630 | 1,340 | 경기도 | 부천시 | 상1동 | 20160704 | 99991231 |
| 106 | 41195680 | 1,340 | 경기도 | 부천시 원미구 | 상1동 | 19930512 | 20160704 |
| 107 | 41190640 | 1,341 | 경기도 | 부천시 | 중4동 | 20160704 | 99991231 |
| 108 | 41195660 | 1,341 | 경기도 | 부천시 원미구 | 중4동 | 19961005 | 20160704 |
| 109 | 41190650 | 1,342 | 경기도 | 부천시 | 약대동 | 20160704 | 99991231 |
| 110 | 41195610 | 1,342 | 경기도 | 부천시 원미구 | 약대동 | 19930115 | 20160704 |
| 111 | 41190660 | 1,343 | 경기도 | 부천시 | 중1동 | 20160704 | 99991231 |
| 112 | 41195630 | 1,343 | 경기도 | 부천시 원미구 | 중1동 | 19930512 | 20160704 |
| 113 | 41190670 | 1,344 | 경기도 | 부천시 | 중2동 | 20160704 | 99991231 |
| 114 | 41195640 | 1,344 | 경기도 | 부천시 원미구 | 중2동 | 19930512 | 20160704 |
| 115 | 41190680 | 1,345 | 경기도 | 부천시 | 중3동 | 20160704 | 99991231 |
| 116 | 41195650 | 1,345 | 경기도 | 부천시 원미구 | 중3동 | 19950220 | 20160704 |
| 117 | 41190690 | 1,346 | 경기도 | 부천시 | 상2동 | 20160704 | 99991231 |
| 118 | 41195690 | 1,346 | 경기도 | 부천시 원미구 | 상2동 | 20030609 | 20160704 |
| 119 | 41190700 | 1,347 | 경기도 | 부천시 | 상3동 | 20160704 | 99991231 |
| 120 | 41195700 | 1,347 | 경기도 | 부천시 원미구 | 상3동 | 20030609 | 20160704 |
| 121 | 41190710 | 1,348 | 경기도 | 부천시 | 심곡본동 | 20160704 | 99991231 |
| 122 | 41197520 | 1,348 | 경기도 | 부천시 소사구 | 심곡본동 | 19940101 | 20160704 |
| 123 | 41190720 | 1,349 | 경기도 | 부천시 | 심곡본1동 | 20160704 | 99991231 |
| 124 | 41197510 | 1,349 | 경기도 | 부천시 소사구 | 심곡본1동 | 19930115 | 20160704 |
| 125 | 41190730 | 1,350 | 경기도 | 부천시 | 송내1동 | 20160704 | 99991231 |
| 126 | 41197590 | 1,350 | 경기도 | 부천시 소사구 | 송내1동 | 19930115 | 20160704 |
| 127 | 41190740 | 1,351 | 경기도 | 부천시 | 송내2동 | 20160704 | 99991231 |
| 128 | 41197600 | 1,351 | 경기도 | 부천시 소사구 | 송내2동 | 19930115 | 20160704 |
| 129 | 41190750 | 1,352 | 경기도 | 부천시 | 소사본동 | 20160704 | 99991231 |
| 130 | 41197535 | 1,352 | 경기도 | 부천시 소사구 | 소사본동 | 20120101 | 20160704 |
| 131 | 41190760 | 1,353 | 경기도 | 부천시 | 소사본3동 | 20160704 | 99991231 |
| 132 | 41197550 | 1,353 | 경기도 | 부천시 소사구 | 소사본3동 | 19930115 | 20160704 |
| 133 | 41190770 | 1,354 | 경기도 | 부천시 | 괴안동 | 20160704 | 99991231 |
| 134 | 41197570 | 1,354 | 경기도 | 부천시 소사구 | 괴안동 | 19930115 | 20160704 |
| 135 | 41190780 | 1,355 | 경기도 | 부천시 | 범박동 | 20160704 | 99991231 |
| 136 | 41197560 | 1,355 | 경기도 | 부천시 소사구 | 범박동 | 19930115 | 20160704 |
| 137 | 41190790 | 1,356 | 경기도 | 부천시 | 역곡3동 | 20160704 | 99991231 |
| 138 | 41197580 | 1,356 | 경기도 | 부천시 소사구 | 역곡3동 | 19930115 | 20160704 |
| 139 | 41190800 | 1,357 | 경기도 | 부천시 | 성곡동 | 20160704 | 99991231 |
| 140 | 41199510 | 1,357 | 경기도 | 부천시 오정구 | 성곡동 | 19930115 | 20160704 |
| 141 | 41190810 | 1,358 | 경기도 | 부천시 | 고강본동 | 20160704 | 99991231 |
| 142 | 41199540 | 1,358 | 경기도 | 부천시 오정구 | 고강본동 | 19930115 | 20160704 |
| 143 | 41190820 | 1,359 | 경기도 | 부천시 | 고강1동 | 20160704 | 99991231 |
| 144 | 41199550 | 1,359 | 경기도 | 부천시 오정구 | 고강1동 | 19930115 | 20160704 |
| 145 | 41190830 | 1,360 | 경기도 | 부천시 | 오정동 | 20160704 | 99991231 |
| 146 | 41199560 | 1,360 | 경기도 | 부천시 오정구 | 오정동 | 19930115 | 20160704 |
| 147 | 41190840 | 1,361 | 경기도 | 부천시 | 원종1동 | 20160704 | 99991231 |
| 148 | 41199520 | 1,361 | 경기도 | 부천시 오정구 | 원종1동 | 19930115 | 20160704 |
| 149 | 41190850 | 1,362 | 경기도 | 부천시 | 원종2동 | 20160704 | 99991231 |
| 150 | 41199530 | 1,362 | 경기도 | 부천시 오정구 | 원종2동 | 19930115 | 20160704 |
| 151 | 41190860 | 1,363 | 경기도 | 부천시 | 신흥동 | 20160704 | 99991231 |
| 152 | 41199570 | 1,363 | 경기도 | 부천시 오정구 | 신흥동 | 19930115 | 20160704 |
| 153 | 41220259 | 1,421 | 경기도 | 평택시 | 청북읍 | 20160728 | 99991231 |
| 154 | 41220350 | 1,421 | 경기도 | 평택시 | 청북면 | 19950510 | 20160728 |
| 155 | 41271520 | 1,452 | 경기도 | 안산시 상록구 | 사1동 | 20021101 | 20170701 |
| 156 | 41271525 | 1,452 | 경기도 | 안산시 상록구 | 사동 | 20170701 | 99991231 |
| 157 | 41271530 | 1,454 | 경기도 | 안산시 상록구 | 사2동 | 20021101 | 20170701 |
| 158 | 41271532 | 1,454 | 경기도 | 안산시 상록구 | 사이동 | 20170701 | 99991231 |
| 159 | 41271535 | 1,456 | 경기도 | 안산시 상록구 | 사3동 | 20070222 | 20170701 |
| 160 | 41271537 | 1,456 | 경기도 | 안산시 상록구 | 해양동 | 20170701 | 99991231 |
| 161 | 41273520 | 1,467 | 경기도 | 안산시 단원구 | 고잔1동 | 20021101 | 20170701 |
| 162 | 41273525 | 1,467 | 경기도 | 안산시 단원구 | 고잔동 | 20170701 | 99991231 |
| 163 | 41273530 | 1,469 | 경기도 | 안산시 단원구 | 고잔2동 | 20021101 | 20170701 |
| 164 | 41273532 | 1,469 | 경기도 | 안산시 단원구 | 중앙동 | 20170701 | 99991231 |
| 165 | 41273550 | 1,474 | 경기도 | 안산시 단원구 | 원곡1동 | 20021101 | 20170701 |
| 166 | 41273555 | 1,474 | 경기도 | 안산시 단원구 | 백운동 | 20170701 | 99991231 |
| 167 | 41273560 | 1,474 | 경기도 | 안산시 단원구 | 원곡2동 | 20021101 | 20170701 |
| 168 | 41273540 | 1,476 | 경기도 | 안산시 단원구 | 원곡본동 | 20021101 | 20170701 |
| 169 | 41273545 | 1,476 | 경기도 | 안산시 단원구 | 원곡동 | 20170701 | 99991231 |
| 170 | 41273565 | 1,476 | 경기도 | 안산시 단원구 | 신길동 | 20170701 | 99991231 |
| 171 | 41360545 | 1,548 | 경기도 | 남양주시 | 다산1동 | 20171218 | 99991231 |
| 172 | 41360560 | 1,548 | 경기도 | 남양주시 | 도농동 | 19950101 | 20171218 |
| 173 | 41360550 | 1,551 | 경기도 | 남양주시 | 지금동 | 19950101 | 20171218 |
| 174 | 41360565 | 1,551 | 경기도 | 남양주시 | 다산2동 | 20171218 | 99991231 |
| 175 | 41390581 | 1,566 | 경기도 | 시흥시 | 군자동 | 19940701 | 99991231 |
| 176 | 41390582 | 1,566 | 경기도 | 시흥시 | 월곶동 | 20140301 | 99991231 |
| 177 | 41390630 | 1,574 | 경기도 | 시흥시 | 연성동 | 19910907 | 99991231 |
| 178 | 41390631 | 1,574 | 경기도 | 시흥시 | 장곡동 | 20140301 | 99991231 |
| 179 | 41450580 | 1,601 | 경기도 | 하남시 | 감북동 | 19890101 | 99991231 |
| 180 | 41450585 | 1,601 | 경기도 | 하남시 | 위례동 | 20151105 | 99991231 |
| 181 | 41450570 | 1,605 | 경기도 | 하남시 | 풍산동 | 19890101 | 99991231 |
| 182 | 41450610 | 1,605 | 경기도 | 하남시 | 미사1동 | 20140605 | 99991231 |
| 183 | 41450620 | 1,605 | 경기도 | 하남시 | 미사2동 | 20140605 | 99991231 |
| 184 | 41461253 | 1,607 | 경기도 | 용인시 처인구 | 모현읍 | 20171211 | 99991231 |
| 185 | 41461310 | 1,607 | 경기도 | 용인시 처인구 | 모현면 | 20051031 | 20171211 |
| 186 | 41461256 | 1,608 | 경기도 | 용인시 처인구 | 이동읍 | 20171211 | 99991231 |
| 187 | 41461330 | 1,608 | 경기도 | 용인시 처인구 | 이동면 | 20051031 | 20171211 |
| 188 | 41480360 | 1,650 | 경기도 | 파주시 | 파평면 | 19960301 | 99991231 |
| 189 | 41480400 | 1,650 | 경기도 | 파주시 | 진동면 | 19960301 | 99991231 |
| 190 | 41570510 | 1,695 | 경기도 | 김포시 | 김포1동 | 19980401 | 20170418 |
| 191 | 41570515 | 1,695 | 경기도 | 김포시 | 김포본동 | 20170418 | 99991231 |
| 192 | 41570520 | 1,702 | 경기도 | 김포시 | 김포2동 | 19980401 | 20170418 |
| 193 | 41570525 | 1,702 | 경기도 | 김포시 | 장기본동 | 20170418 | 99991231 |
| 194 | 41570570 | 1,702 | 경기도 | 김포시 | 구래동 | 20131028 | 99991231 |
| 195 | 41570580 | 1,702 | 경기도 | 김포시 | 운양동 | 20150202 | 99991231 |
| 196 | 41590262 | 1,706 | 경기도 | 화성시 | 남양읍 | 20141020 | 99991231 |
| 197 | 41590510 | 1,706 | 경기도 | 화성시 | 남양동 | 20010321 | 20141020 |
| 198 | 41590420 | 1,727 | 경기도 | 화성시 | 동탄면 | 20010321 | 20180122 |
| 199 | 41590588 | 1,727 | 경기도 | 화성시 | 동탄4동 | 20150102 | 99991231 |
| 200 | 41610360 | 1,735 | 경기도 | 광주시 | 중부면 | 20010321 | 20151016 |
| 201 | 41610370 | 1,735 | 경기도 | 광주시 | 남한산성면 | 20151016 | 99991231 |
| 202 | 41670250 | 1,764 | 경기도 | 여주시 | 가남읍 | 20130923 | 99991231 |
| 203 | 41730320 | 1,764 | 경기도 | 여주군 | 가남면 | 19880423 | 20130923 |
| 204 | 41670310 | 1,765 | 경기도 | 여주시 | 점동면 | 20130923 | 99991231 |
| 205 | 41730310 | 1,765 | 경기도 | 여주군 | 점동면 | 19880423 | 20130923 |
| 206 | 41670320 | 1,766 | 경기도 | 여주시 | 흥천면 | 20130923 | 99991231 |
| 207 | 41730330 | 1,766 | 경기도 | 여주군 | 흥천면 | 19880423 | 20130923 |
| 208 | 41670330 | 1,767 | 경기도 | 여주시 | 금사면 | 20130923 | 99991231 |
| 209 | 41730340 | 1,767 | 경기도 | 여주군 | 금사면 | 19880423 | 20130923 |
| 210 | 41670340 | 1,768 | 경기도 | 여주시 | 능서면 | 20130923 | 99991231 |
| 211 | 41730350 | 1,768 | 경기도 | 여주군 | 능서면 | 19880423 | 20130923 |
| 212 | 41670350 | 1,769 | 경기도 | 여주시 | 대신면 | 20130923 | 99991231 |
| 213 | 41730360 | 1,769 | 경기도 | 여주군 | 대신면 | 19880423 | 20130923 |
| 214 | 41670360 | 1,770 | 경기도 | 여주시 | 북내면 | 20130923 | 99991231 |
| 215 | 41730370 | 1,770 | 경기도 | 여주군 | 북내면 | 19880423 | 20130923 |
| 216 | 41670370 | 1,771 | 경기도 | 여주시 | 강천면 | 20130923 | 99991231 |
| 217 | 41730380 | 1,771 | 경기도 | 여주군 | 강천면 | 19880423 | 20130923 |
| 218 | 41670380 | 1,772 | 경기도 | 여주시 | 산북면 | 20130923 | 99991231 |
| 219 | 41730390 | 1,772 | 경기도 | 여주군 | 산북면 | 19890401 | 20130923 |
| 220 | 41670510 | 1,774 | 경기도 | 여주시 | 여흥동 | 20130923 | 99991231 |
| 221 | 41670520 | 1,774 | 경기도 | 여주시 | 중앙동 | 20130923 | 99991231 |
| 222 | 41670530 | 1,774 | 경기도 | 여주시 | 오학동 | 20130923 | 99991231 |
| 223 | 41730250 | 1,774 | 경기도 | 여주군 | 여주읍 | 19880423 | 20130923 |
| 224 | 41820340 | 1,801 | 경기도 | 가평군 | 하면 | 19880423 | 20151216 |
| 225 | 41820345 | 1,801 | 경기도 | 가평군 | 조종면 | 20151216 | 99991231 |
| 226 | 42750370 | 1,952 | 강원도 | 영월군 | 수주면 | 19880423 | 20161115 |
| 227 | 42750380 | 1,952 | 강원도 | 영월군 | 무릉도원면 | 20161115 | 99991231 |
| 228 | 42780253 | 1,976 | 강원도 | 철원군 | 김화읍 | 19880423 | 99991231 |
| 229 | 42780330 | 1,976 | 강원도 | 철원군 | 근북면 | 19930310 | 99991231 |
| 230 | 43111310 | 2,009 | 충청북도 | 청주시 상당구 | 낭성면 | 20140701 | 99991231 |
| 231 | 43710310 | 2,009 | 충청북도 | 청원군 | 낭성면 | 19880423 | 20140701 |
| 232 | 43111320 | 2,010 | 충청북도 | 청주시 상당구 | 미원면 | 20140701 | 99991231 |
| 233 | 43710320 | 2,010 | 충청북도 | 청원군 | 미원면 | 19880423 | 20140701 |
| 234 | 43111330 | 2,011 | 충청북도 | 청주시 상당구 | 가덕면 | 20140701 | 99991231 |
| 235 | 43710330 | 2,011 | 충청북도 | 청원군 | 가덕면 | 19880423 | 20140701 |
| 236 | 43111340 | 2,012 | 충청북도 | 청주시 상당구 | 남일면 | 20140701 | 99991231 |
| 237 | 43710340 | 2,012 | 충청북도 | 청원군 | 남일면 | 19880423 | 20140701 |
| 238 | 43111350 | 2,013 | 충청북도 | 청주시 상당구 | 문의면 | 20140701 | 99991231 |
| 239 | 43710360 | 2,013 | 충청북도 | 청원군 | 문의면 | 19880423 | 20140701 |
| 240 | 43112310 | 2,027 | 충청북도 | 청주시 서원구 | 남이면 | 20140701 | 99991231 |
| 241 | 43710350 | 2,027 | 충청북도 | 청원군 | 남이면 | 19880423 | 20140701 |
| 242 | 43112320 | 2,028 | 충청북도 | 청주시 서원구 | 현도면 | 20140701 | 99991231 |
| 243 | 43710370 | 2,028 | 충청북도 | 청원군 | 현도면 | 19880423 | 20140701 |
| 244 | 43112510 | 2,029 | 충청북도 | 청주시 서원구 | 사직1동 | 20140701 | 99991231 |
| 245 | 43113630 | 2,029 | 충청북도 | 청주시 흥덕구 | 사직제1동 | 19950101 | 20140701 |
| 246 | 43112520 | 2,030 | 충청북도 | 청주시 서원구 | 사직2동 | 20140701 | 99991231 |
| 247 | 43113640 | 2,030 | 충청북도 | 청주시 흥덕구 | 사직제2동 | 19950101 | 20140701 |
| 248 | 43112530 | 2,031 | 충청북도 | 청주시 서원구 | 사창동 | 20140701 | 99991231 |
| 249 | 43113650 | 2,031 | 충청북도 | 청주시 흥덕구 | 사창동 | 19950101 | 20140701 |
| 250 | 43112540 | 2,032 | 충청북도 | 청주시 서원구 | 모충동 | 20140701 | 99991231 |
| 251 | 43113660 | 2,032 | 충청북도 | 청주시 흥덕구 | 모충동 | 19950101 | 20140701 |
| 252 | 43112550 | 2,033 | 충청북도 | 청주시 서원구 | 산남동 | 20140701 | 99991231 |
| 253 | 43113722 | 2,033 | 충청북도 | 청주시 흥덕구 | 산남동 | 20080101 | 20140701 |
| 254 | 43112560 | 2,034 | 충청북도 | 청주시 서원구 | 분평동 | 20140701 | 99991231 |
| 255 | 43113723 | 2,034 | 충청북도 | 청주시 흥덕구 | 분평동 | 20080101 | 20140701 |
| 256 | 43112570 | 2,035 | 충청북도 | 청주시 서원구 | 수곡1동 | 20140701 | 99991231 |
| 257 | 43113727 | 2,035 | 충청북도 | 청주시 흥덕구 | 수곡1동 | 19960101 | 20140701 |
| 258 | 43112580 | 2,036 | 충청북도 | 청주시 서원구 | 수곡2동 | 20140701 | 99991231 |
| 259 | 43113728 | 2,036 | 충청북도 | 청주시 흥덕구 | 수곡2동 | 19960101 | 20140701 |
| 260 | 43112590 | 2,037 | 충청북도 | 청주시 서원구 | 성화.개신.죽림동 | 20140701 | 99991231 |
| 261 | 43113780 | 2,037 | 충청북도 | 청주시 흥덕구 | 성화.개신.죽림동 | 19950101 | 20140701 |
| 262 | 43113250 | 2,038 | 충청북도 | 청주시 흥덕구 | 오송읍 | 20140701 | 99991231 |
| 263 | 43710256 | 2,038 | 충청북도 | 청원군 | 오송읍 | 20120101 | 20140701 |
| 264 | 43113310 | 2,039 | 충청북도 | 청주시 흥덕구 | 강내면 | 20140701 | 99991231 |
| 265 | 43710390 | 2,039 | 충청북도 | 청원군 | 강내면 | 19880423 | 20140701 |
| 266 | 43113320 | 2,040 | 충청북도 | 청주시 흥덕구 | 옥산면 | 20140701 | 99991231 |
| 267 | 43710410 | 2,040 | 충청북도 | 청원군 | 옥산면 | 19880423 | 20140701 |
| 268 | 43114250 | 2,058 | 충청북도 | 청주시 청원구 | 내수읍 | 20140701 | 99991231 |
| 269 | 43710250 | 2,058 | 충청북도 | 청원군 | 내수읍 | 20000101 | 20140701 |
| 270 | 43114253 | 2,059 | 충청북도 | 청주시 청원구 | 오창읍 | 20140701 | 99991231 |
| 271 | 43710253 | 2,059 | 충청북도 | 청원군 | 오창읍 | 20070101 | 20140701 |
| 272 | 43114310 | 2,060 | 충청북도 | 청주시 청원구 | 북이면 | 20140701 | 99991231 |
| 273 | 43710430 | 2,060 | 충청북도 | 청원군 | 북이면 | 19880423 | 20140701 |
| 274 | 43111580 | 2,061 | 충청북도 | 청주시 상당구 | 우암동 | 19950101 | 20140701 |
| 275 | 43114510 | 2,061 | 충청북도 | 청주시 청원구 | 우암동 | 20140701 | 99991231 |
| 276 | 43111590 | 2,062 | 충청북도 | 청주시 상당구 | 내덕제1동 | 19950101 | 20140701 |
| 277 | 43114520 | 2,062 | 충청북도 | 청주시 청원구 | 내덕1동 | 20140701 | 99991231 |
| 278 | 43111600 | 2,063 | 충청북도 | 청주시 상당구 | 내덕제2동 | 19950101 | 20140701 |
| 279 | 43114530 | 2,063 | 충청북도 | 청주시 청원구 | 내덕2동 | 20140701 | 99991231 |
| 280 | 43111610 | 2,064 | 충청북도 | 청주시 상당구 | 율량.사천동 | 19950101 | 20140701 |
| 281 | 43114540 | 2,064 | 충청북도 | 청주시 청원구 | 율량.사천동 | 20140701 | 99991231 |
| 282 | 43111790 | 2,065 | 충청북도 | 청주시 상당구 | 오근장동 | 19950101 | 20140701 |
| 283 | 43114550 | 2,065 | 충청북도 | 청주시 청원구 | 오근장동 | 20140701 | 99991231 |
| 284 | 43130380 | 2,074 | 충청북도 | 충주시 | 가금면 | 19950101 | 20140201 |
| 285 | 43130385 | 2,074 | 충청북도 | 충주시 | 중앙탑면 | 20140201 | 99991231 |
| 286 | 43150517 | 2,102 | 충청북도 | 제천시 | 의암동 | 20110101 | 20170101 |
| 287 | 43150518 | 2,102 | 충청북도 | 제천시 | 의림지동 | 20170101 | 99991231 |
| 288 | 43150527 | 2,104 | 충청북도 | 제천시 | 인성동 | 20110101 | 20170101 |
| 289 | 43150528 | 2,104 | 충청북도 | 제천시 | 중앙동 | 20170101 | 99991231 |
| 290 | 44133560 | 2,219 | 충청남도 | 천안시 서북구 | 백석동 | 20080623 | 99991231 |
| 291 | 44133565 | 2,219 | 충청남도 | 천안시 서북구 | 불당동 | 20131014 | 99991231 |
| 292 | 44133570 | 2,221 | 충청남도 | 천안시 서북구 | 부성동 | 20080623 | 20131014 |
| 293 | 44133580 | 2,221 | 충청남도 | 천안시 서북구 | 부성1동 | 20131014 | 99991231 |
| 294 | 44133590 | 2,221 | 충청남도 | 천안시 서북구 | 부성2동 | 20131014 | 99991231 |
| 295 | 44800256 | 2,371 | 충청남도 | 홍성군 | 홍북읍 | 20170801 | 99991231 |
| 296 | 44800310 | 2,371 | 충청남도 | 홍성군 | 홍북면 | 19880423 | 20170801 |
| 297 | 45710256 | 2,557 | 전라북도 | 완주군 | 용진읍 | 20151001 | 99991231 |
| 298 | 45710310 | 2,557 | 전라북도 | 완주군 | 용진면 | 19880423 | 20151001 |
| 299 | 46170390 | 2,736 | 전라남도 | 나주시 | 금천면 | 19950101 | 99991231 |
| 300 | 46170620 | 2,736 | 전라남도 | 나주시 | 빛가람동 | 20140224 | 99991231 |
| 301 | 47150320 | 3,013 | 경상북도 | 김천시 | 남면 | 19950101 | 99991231 |
| 302 | 47150640 | 3,013 | 경상북도 | 김천시 | 율곡동 | 20140102 | 99991231 |
| 303 | 47830250 | 3,212 | 경상북도 | 고령군 | 고령읍 | 19881001 | 20150402 |
| 304 | 47830253 | 3,212 | 경상북도 | 고령군 | 대가야읍 | 20150402 | 99991231 |
| 305 | 47900320 | 3,250 | 경상북도 | 예천군 | 상리면 | 19880423 | 20160201 |
| 306 | 47900420 | 3,250 | 경상북도 | 예천군 | 효자면 | 20160201 | 99991231 |
| 307 | 47900330 | 3,251 | 경상북도 | 예천군 | 하리면 | 19880423 | 20160201 |
| 308 | 47900430 | 3,251 | 경상북도 | 예천군 | 은풍면 | 20160201 | 99991231 |
| 309 | 47930320 | 3,272 | 경상북도 | 울진군 | 서면 | 19880423 | 20150421 |
| 310 | 47930390 | 3,272 | 경상북도 | 울진군 | 금강송면 | 20150421 | 99991231 |
| 311 | 47930340 | 3,273 | 경상북도 | 울진군 | 원남면 | 19880423 | 20150421 |
| 312 | 47930400 | 3,273 | 경상북도 | 울진군 | 매화면 | 20150421 | 99991231 |
| 313 | 48125550 | 3,302 | 경상남도 | 창원시 마산합포구 | 반월동 | 20100701 | 20170101 |
| 314 | 48125560 | 3,302 | 경상남도 | 창원시 마산합포구 | 중앙동 | 20100701 | 20170101 |
| 315 | 48125565 | 3,302 | 경상남도 | 창원시 마산합포구 | 반월중앙동 | 20170101 | 99991231 |
| 316 | 48125590 | 3,309 | 경상남도 | 창원시 마산합포구 | 동서동 | 20100701 | 20170101 |
| 317 | 48125600 | 3,309 | 경상남도 | 창원시 마산합포구 | 성호동 | 20100701 | 20170101 |
| 318 | 48125630 | 3,309 | 경상남도 | 창원시 마산합포구 | 오동동 | 20100701 | 99991231 |
| 319 | 48127530 | 3,317 | 경상남도 | 창원시 마산회원구 | 석전1동 | 20100701 | 20170101 |
| 320 | 48127540 | 3,317 | 경상남도 | 창원시 마산회원구 | 석전2동 | 20100701 | 20170101 |
| 321 | 48127545 | 3,317 | 경상남도 | 창원시 마산회원구 | 석전동 | 20170101 | 99991231 |
| 322 | 48170510 | 3,358 | 경상남도 | 진주시 | 망경동 | 19880423 | 20130501 |
| 323 | 48170515 | 3,358 | 경상남도 | 진주시 | 천전동 | 20130501 | 99991231 |
| 324 | 48170520 | 3,358 | 경상남도 | 진주시 | 강남동 | 19880423 | 20130501 |
| 325 | 48170530 | 3,358 | 경상남도 | 진주시 | 칠암동 | 19880423 | 20130501 |
| 326 | 48170545 | 3,362 | 경상남도 | 진주시 | 성지동 | 19970701 | 20130501 |
| 327 | 48170555 | 3,362 | 경상남도 | 진주시 | 성북동 | 20130501 | 99991231 |
| 328 | 48170575 | 3,362 | 경상남도 | 진주시 | 봉안동 | 19970701 | 20130501 |
| 329 | 48170565 | 3,363 | 경상남도 | 진주시 | 중앙동 | 19970701 | 99991231 |
| 330 | 48170615 | 3,363 | 경상남도 | 진주시 | 봉수동 | 19970701 | 20130501 |
| 331 | 48170645 | 3,363 | 경상남도 | 진주시 | 옥봉동 | 19970701 | 20130501 |
| 332 | 48170590 | 3,366 | 경상남도 | 진주시 | 상봉동동 | 19900501 | 20130501 |
| 333 | 48170595 | 3,366 | 경상남도 | 진주시 | 상봉동 | 20130501 | 99991231 |
| 334 | 48170600 | 3,366 | 경상남도 | 진주시 | 상봉서동 | 19900501 | 20130501 |
| 335 | 48170660 | 3,372 | 경상남도 | 진주시 | 상대1동 | 19880423 | 20170101 |
| 336 | 48170670 | 3,372 | 경상남도 | 진주시 | 상대2동 | 19880423 | 20170101 |
| 337 | 48170673 | 3,372 | 경상남도 | 진주시 | 상대동 | 20170101 | 99991231 |
| 338 | 48170676 | 3,375 | 경상남도 | 진주시 | 하대1동 | 19970701 | 20170201 |
| 339 | 48170677 | 3,375 | 경상남도 | 진주시 | 하대2동 | 19970701 | 20170201 |
| 340 | 48170678 | 3,375 | 경상남도 | 진주시 | 하대동 | 20170201 | 99991231 |
| 341 | 48170250 | 3,383 | 경상남도 | 진주시 | 문산읍 | 19950302 | 99991231 |
| 342 | 48170410 | 3,383 | 경상남도 | 진주시 | 금산면 | 19950101 | 99991231 |
| 343 | 48170750 | 3,383 | 경상남도 | 진주시 | 충무공동 | 20131218 | 99991231 |
| 344 | 48250310 | 3,430 | 경상남도 | 김해시 | 장유면 | 19950510 | 20130701 |
| 345 | 48250610 | 3,430 | 경상남도 | 김해시 | 장유1동 | 20130701 | 99991231 |
| 346 | 48250620 | 3,430 | 경상남도 | 김해시 | 장유2동 | 20130701 | 99991231 |
| 347 | 48250630 | 3,430 | 경상남도 | 김해시 | 장유3동 | 20130701 | 99991231 |
| 348 | 48310510 | 3,458 | 경상남도 | 거제시 | 장승포동 | 19950101 | 99991231 |
| 349 | 48310520 | 3,458 | 경상남도 | 거제시 | 마전동 | 19950101 | 20160501 |
| 350 | 48730253 | 3,495 | 경상남도 | 함안군 | 칠원읍 | 20150101 | 99991231 |
| 351 | 48730370 | 3,495 | 경상남도 | 함안군 | 칠원읍 | 19880423 | 20150101 |
